# Combination of CAD/CAM technologies and conventional processing in the fabrication of a maxillary obturator prosthesis: a clinical report

**DOI:** 10.25122/jml-2024-0017

**Published:** 2024-03

**Authors:** Ines Saadellaoui, Sana Bekri, Amel Labidi, Mohamed ben Yaala, Yosra Mabrouk, Lamia Mansour

**Affiliations:** 1 Department of Removable Prosthodontics, Approche Biologique et Clinique Dento-Faciale (LR12ES10) Laboratory, Faculty of Dental Medicine, University of Monastir, Monastir, Tunisia

**Keywords:** squamous cell carcinoma, optical impression, obturator, prosthesis, computer-aided design, computer-aided manufacturing, selective laser melting, selective laser sintering

## Abstract

Soft and hard tissue defects resulting from resective surgeries for carcinomas located in the maxillary arches can cause functional, esthetic, and psychological damage. A removable obturator prosthesis offers several advantages, restoring oral functions and improving patients’ quality of life. Technological advancements, such as the use of intraoral scanning and computer-aided design (CAD) and manufacturing, reduce laboratory working time, eliminate the risk of impression material aspiration, and address challenges related to whole tissue undercut impression. Here, we report the case of a partially edentulous female patient with a velo-palatal defect for whom a rigid maxillary obturator prosthesis was fabricated. Digital impressions were taken and the standard tessellation language files of the scans were sent to the laboratory. Using dental CAD software, the maxillary metallic framework was designed and manufactured using selective laser melting technology. The obturators and artificial teeth were conventionally processed, with acrylic resin used for the rigid obturators. The resulting obturator prosthesis made it possible to close the oro-nasal communication and to improve swallowing, speaking, and chewing.

## INTRODUCTION

The maxilla plays a primordial role in mastication, deglutition, and phonation. Maxillary defects can be the result of congenital factors, trauma, osteonecrosis, or tumor pathology [1]. The most common malignant tumors of the upper aerodigestive tract are squamous cell carcinomas [2]. These pathologies require partial or complete maxillectomy, leaving the patient with an oronasal defect that negatively affects mastication, phonation (leading to hypernasal speech), swallowing, and psychological well-being [3–5].

The treatment of post-maxillectomy defects includes different options, such as reconstructive surgery or rehabilitation with an obturator prosthesis, with the choice depending on the specific clinical situation. Although surgical free flap reconstruction remains a valid alternative, its use can be limited by factors such as the risk of complications, systemic pathologies, the possibility of failure, and patient refusal [6].

An obturator prosthesis is a device used to close a congenital or acquired tissue opening, primarily of the hard palate and/or contiguous alveolar structures [4]. The history of obturator prosthesis is well documented. Ambroise Paré was the first to use an artificial device to close a palatal defect as early as the 1500s [4]. Claude Martin described the use of a surgical obturator prosthesis in 1875, and Fry described the use of impressions before surgery in 1927 [4].

For edentulous patients with maxillofacial defects, the fabrication of an obturator prosthesis can be done conventionally or through a digital workflow. The progressive improvements of digital technologies applied in the field of dentistry and maxillo-facial surgery, such as computer-aided design (CAD) and computer-aided manufacturing (CAM), have been successfully applied in dentistry and maxillofacial surgery, enhancing the design and fabrication of obturator prostheses [7–10].

Here, we present the management of a velo-palatal defect in a partially edentulous patient using a rigid obturator prosthesis that incorporates CAD/CAM technologies alongside conventional processing techniques.

## CASE PRESENTATION

A 61-year-old female patient consulted the Department of Prosthodontics seeking for oral rehabilitation to address functional concerns. Her medical history revealed a diagnosis of squamous cell carcinoma of the palate, which had been treated with surgical resection and head and neck radiotherapy 2 years prior. The patient complained of chewing and phonation difficulties associated with nasal leakage of fluids.

### Clinical findings and diagnostic assessment

**Figure 1:**
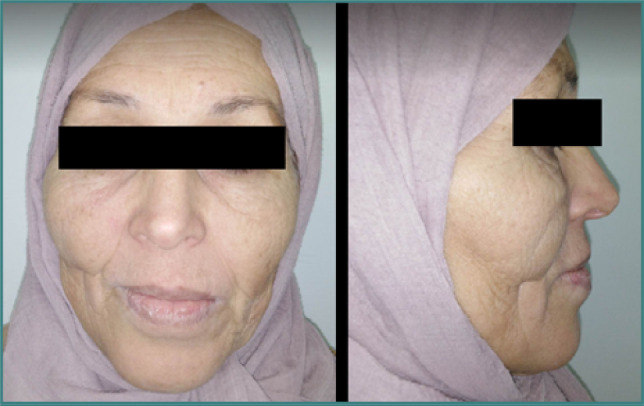
Extra-oral views of the patient

**Figure 2:**
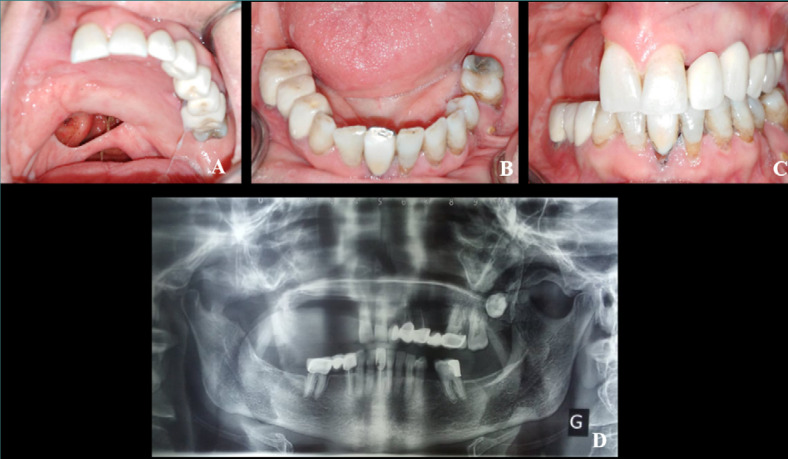
Initial situation. A–C, Intra oral views of the maxillary arch, velo-palatal defect, and mandibular arch. D, Panoramic X-ray.

The extra-oral examination showed symmetry in the levels of the face and a sagging of the right half-face. The right corner of the lip was convergent relative to the bipupillary line (Figure 1). The patient had a moderately sufficient mouth opening and a straight mouth opening/closing path. The intra-oral examination revealed a maxillary defect involving the right palatine bone, the soft palate, and the upper right alveolar arch, resulting in an oronasal communication (Figure 2A). According to Aramany’s classification of maxillary defects [11], this was a class II defect. Based on Benoist’s classification of soft palate defects, it was a divided velum associated with partial edentulism (class IC) [12].

A total of eight maxillary teeth remained, from the right central incisor (tooth 11) to the second left molar (tooth 27). In the mandibular arch, all teeth were present except for 36, 47, 48, and 38 (Figure 2A–C). The osteomucosal-bearing surface showed a flat palate and an extension of the mucous membrane covering the inner side of the cheek to the level of the right edentulous ridge. Saliva examination showed a remarkable viscosity. Radiological examination indicated that the crown-to-root ratio was 1 for all maxillary and mandibular teeth (Figure 2D). The examination of oral functions revealed swallowing and chewing difficulties, as well as the presence of rhinolalia.

### Therapeutic intervention

The oral cavity sanitation phase included oral hygiene measures and caries curettage on tooth 11.

Based on data from the clinical and radiological examinations, a maxillary metallic framework obturator prosthesis was indicated, consisting of a palatal plate, a rigid palatal obturator, and a rigid velar obturator.

**Figure 3:**
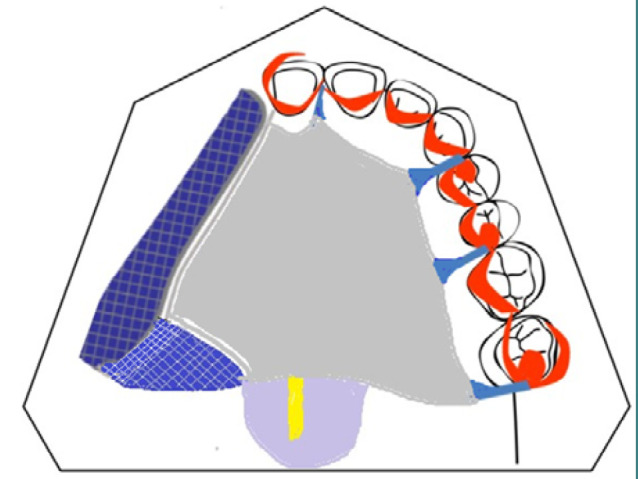
Framework layout

A frame layout was established with clasps strategically placed in accordance with the topography of the edentulism. The number of occlusal and cingulate supports and coronal bars was increased to compensate for the lack of dental and osteo-mucosal support surfaces owing to hard and soft tissues defects. A mesh saddle was placed at the level of the edentulous sector and the palatal defect. In addition, a metal tutor connected the palatal plate to the velar obturator, also serving as a supporting framework for the obturator (Figure 3).

**Figure 4:**
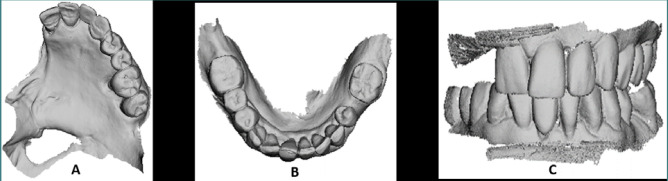
A–C, Digital scans of the maxillary arch and palatal defect (A), mandibular arch (B), and of the occlusion (C)

The prosthetic procedures began with enameloplasties related to the already established maxillary frame layout. The maxillary arch and palate, maxillary defect, mandibular arch, and occlusal relationships were scanned using a TRIOS intra-oral optical scanner (3Shape) (Figure 4A–C). The 3D images were exported as standard tessellation language (STL) files and merged to form a 3D digital cast of the maxillary defect containing the anatomic structures needed for the maxillary prosthesis. This included the defect cavity, maxillary dentition, and the palate. A 3D digital cast of the mandibular arch was also obtained.

**Figure 5:**
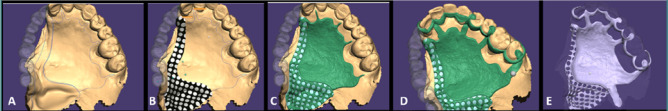
Digital framework design. A–C, Tracing the limits of the palatal plate and the saddle. D, Establishment of occlusal rests and connections. E, Digital design of the tutor.

**Figure 6:**
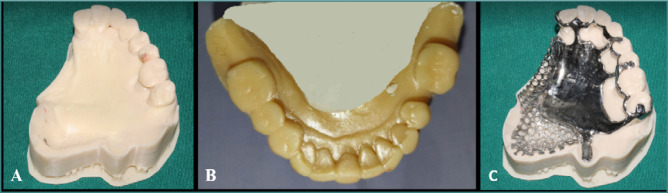
3D impression technique. A,B, Physical resin casts. C, Metallic framework in cobalt chromium alloy.

The digital design of the framework was performed using Exocad software, respecting the insertion axis. Undercut areas were filled, and the limits of the major connector and saddle were defined (Figure 5A–C). This was followed by the establishment of occlusal rests and connections (Figure 5D). The tutor, which required an oblique orientation, was also designed (Figure 5E). After completing the design, physical resin casts were produced using the 3D impression technique (Figure 6A,B). The maxillary framework was printed in cobalt–chromium alloy using the selective laser melting technique (Figure 6C).

**Figure 7:**
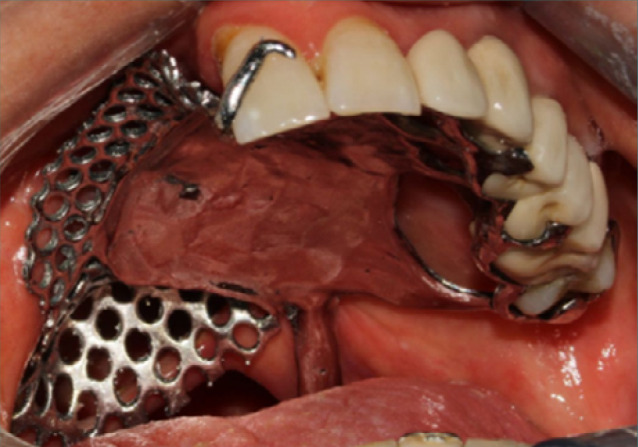
Fitting of the metallic framework in the mouth

**Figure 8:**
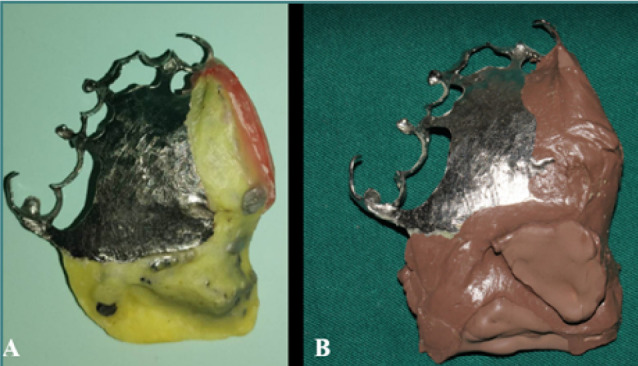
Anatomo-functional impression. A, Equipment of the saddle with Formatray resin. B, Anatomo-functional impression with polysulfide impression material.

The printed metal framework was placed and adjusted intra-orally (Figure 7). The terminal saddle as well as the obturators’ support saddle were equipped with Formatray resin (Kerr) and adjusted in the mouth (Figure 8A).

At this stage, the digital workflow was paused, and an anatomo-functional impression was made using polysulfide impression material. This impression captured both the palatal and velar defects, as well as the limits of the peripheral musculature’s action. The patient was instructed to perform head rotation movements to the right and left, as well as extension forwards and backwards, while pronouncing the prolonged letter ‘A’ (Figure 8B).

**Figure 9:**
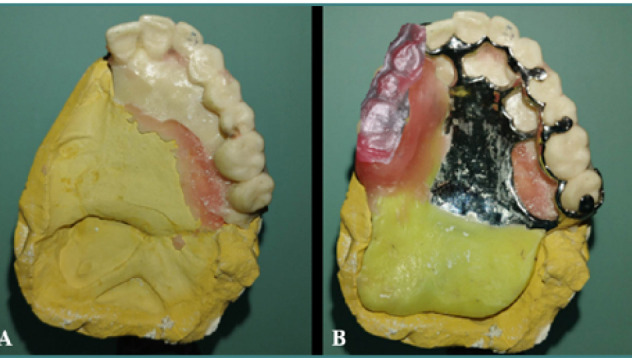
Occlusion recording. A, Corrected cast. B, Equipment of the saddle with an occlusion rim.

**Figure 10:**
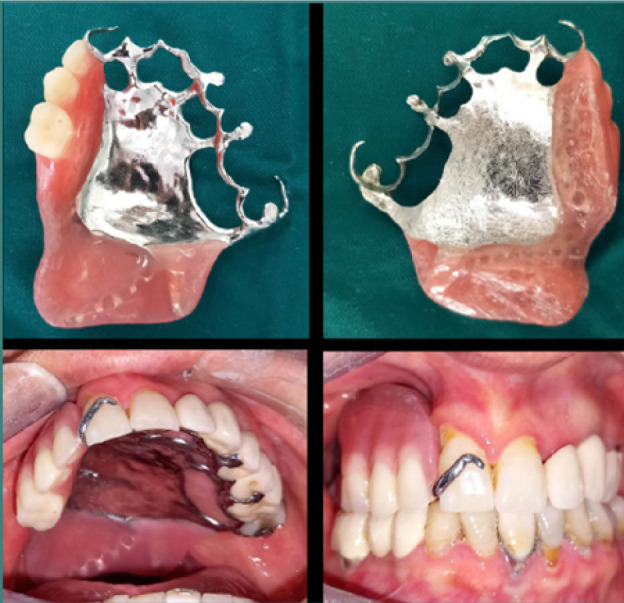
Maxillary obturator prosthesis

The cast resulting from the digital impression was cut, preserving the toothed sector. The metallic frameworks were then set up, and the impression was boxed for casting. This process yielded a corrected cast, which consisted of a toothed sector from the digital impression and a toothless sector from the anatomo-functional impression (Figure 9A). The saddle was then equipped with an occlusion rim (Figure 9B). Occlusal relationships were recorded in centric relation and correct occlusal vertical dimension. Resin artificial teeth were mounted on wax, with the shape, shade, and dimensions chosen to match the residual teeth and the available prosthetic space. After fitting in the mouth, the resin was polymerized, and the obturator prosthesis was placed in the mouth (Figure 10).

### Follow-up and outcomes

The patient was satisfied with the final result. Recommendations concerning the maintenance of the prosthesis were provided, which specified that the prosthesis must be brushed after each meal with a soft toothbrush, immersed in a solution of chlorhexidine digluconate for 15 min daily, and rinsed thoroughly after using these products.

Periodic check-ups were scheduled to monitor the stability of the maxillary obturator prosthesis and to detect any complications or mucosal irritations. If complications occur, they should be promptly treated, and their healing should be carefully monitored.

## DISCUSSION

The obturator prosthesis is a reliable treatment option that offers several advantages. It is simple, non-surgical, and effectively eliminates oro-nasal and oro-antral communications, resulting in the restoration of adequate oral functions, improved esthetics, and enhanced quality of life [3].

An accurate impression is essential for the success of the obturator prosthesis. The use of an optical scanner has significant benefits, such as preventing the aspiration of impression material into the defect and overcoming the difficulties associated with whole tissue undercut impression [13].

Compared to the conventional impressions, digital impressions can save chair-time, laboratory working time, and the amount of dental materials required [3,14,15]. In this clinical case, 2 min were sufficient to complete both the digital impressions and the jaw relationship recording.

Patients who tend to gag during impression taking, those with allergies to impression materials, and those with special needs or anxiety may tolerate intra-oral scanning better than conventional impressions. In addition, using an optical scanner with suitable dimensions can avoid the difficulties of handling impression trays in the case of patients with limited mouth opening due to scarring or radiation therapy [15–18]. In the present case, the patient reported that “the impression taking step was simple, quick, and comfortable”. Another advantage of digital impressions is improved communication between the dental laboratory and the dental office through the use of screenshots [3].

However, some limitations of intra-oral scanners should be noted. Digital scanning can only develop a mucostatic impression by capturing tissues in a passive state. Soft tissue movement can disrupt the scanning process, altering the morphology of the site, the appropriate prosthesis border extension, and the peripheral seal. In addition, in case of large distal extension edentulism, determining the difference in compressibility between the fibromucosa and the periodontal ligament can be challenging [7]. In this clinical case, it was difficult to capture the fibromucosa in a functional state using an optical scanner, which explains the combination of conventional and digital impression techniques, using dynamic impressions around the obturator and movable tissues to reproduce the dynamic state.

The digital design of removable prostheses can be carried out using geometric analysis tools, which create designs of micrometer-level accuracy that can be viewed in cross-section. This allows to approve and modify the design before the framework is fabricated. The subsequent fabrication steps include several advanced manufacturing processes, which can be subcategorized into two groups: subtractive manufacturing, which involves removing material from a solid block to create the desired shape (such as milling with the use of burs, disks, or lasers), and additive manufacturing, which builds the prosthesis layer by layer [19].

Both milling and printing of frameworks have advantages and disadvantages. Both methods offer shorter production times of a few hours compared to conventional techniques, which can take days for manufacture [20]. Milling offers advantages such as reduced manufacturing deficiencies, including porosities, polymerization shrinkage, and inhomogeneous consistencies. The latter can be avoided as the prosthesis is milled from a consistent block of the same material [19,21]. Although milling is commonly used in fixed prosthodontics, it is less used in removable prosthodontics owing to the complex shapes and varying thicknesses of the components of removable partial dentures (RPDs). In addition, milling does not provide the accuracy offered by laser sintering, as cutting tools have specific thickness limitations, posing accuracy constraints [19].

Additive techniques, such as selective laser melting and selective laser sintering, offer relatively low costs for both machines and materials compared to other fabrication techniques. However, the main concern with these techniques is polymerization shrinkage. Although these methods have been reported to provide excellent fit and satisfy patients, laboratory studies are needed to evaluate the extent of shrinkage [22]. Despite the potential for shrinkage, these techniques may reduce errors compared to conventional fabrication methods. Clinically acceptable fit has been reported for RPD frameworks fabricated with digital technology [3]. Maryod and Taha found digitally fabricated RPDs to have greater retention and fit than those produced using conventional methods in 20 patients with mandibular Kennedy class I RPDs [23]. Other studies have shown that digitally fabricated RPDs have a significantly better retention and fit compared to the conventional RPDs, with tests performed at different intervals over a 3-month follow-up period [24]. Authors have also reported improved mechanical properties and higher patient satisfaction in terms of denture cleaning, speaking, mastication, and comfort. The availability of saved data for future prosthesis reproduction is another advantage of digital techniques [14].

In this clinical report, the framework was the CAD/CAM portion, whereas the rigid obturators and artificial teeth were conventionally processed. The metallic framework was created using the selective laser melting technique. This process involves applying metallic powder materials that are free of binders and fluxing agents, then heating the powder to its melting temperature with a laser beam so that the layer of the metallic powder is fully molten [25].

Artificial teeth can also be printed or milled. Printed teeth often lack a variety of shapes and shades, whereas milled teeth offer a wide variety of options that can be selected by the patient and clinician [22].

In this clinical case, the palatal and velar obturators were rigid and made of methyl methacrylate. This material has the advantage of being durable in the long term, it can be easily cleaned, and it is perfectly polished [26].

The velar obturator should meet specific criteria for optimal function. Its superior surface is made convex and polished to facilitate the deflection of nasal secretions, whereas its inferior surface is made slightly concave to prevent tongue interference. The lateral margins are polished to improve hygiene and deflection of secretions, and the bulb has a superior extension of around 8 mm. The lateral dimension is determined by the movements of the lateral and posterior pharyngeal wall during border molding [27,28].

Another type of velar obturator described in the literature includes a removable denture featuring a thick dental dam that serves as a membrane obturator. Mimetic restoration of the velar anatomy using a membrane obturator is a promising technique for restoring speech and swallowing and improving the quality of life of patients with acquired soft palate defects [29,30].

It is well known that irradiated tissues are brittle, characterized by hypovascularization, hypocellularity, and hypoxia. Consequently, irradiated patients are more susceptible to the development of viral and bacterial infections and dental decays that are often asymptomatic and rapidly progressing [31]. Therefore, regular monitoring of irradiated patients is necessary. Maintaining oral hygiene and daily cleaning of the prosthesis are crucial prophylactic measures for these patients.

## CONCLUSION

The maxillary obturator prosthesis improved oral functions, enhanced patient comfort, and closed the oro-nasal communication. The functional integration of the prosthesis is based on the adequate exploitation of the dental and osteo-mucosal support surfaces. Digital impressions ensure precise recording of these surfaces as well as the defect boundaries.

In cases of large terminal edentulism, digital impressions alone may not adequately capture the tissue duality characteristic of extensive edentulous areas. Therefore, a combination of conventional and digital techniques is indispensable.

It is also important to note that close monitoring and long-term follow-ups are essential for managing maxillectomy defects and ensuring the stability of the maxillary obturator prosthesis.
